# Prevalence and associated factors of erectile dysfunction in men with type 2 diabetes mellitus in eastern Sudan

**DOI:** 10.1186/s12902-022-01060-0

**Published:** 2022-05-28

**Authors:** Saeed M. Omar, Imad R. Musa, Maysoon B. Idrees, Omer Abdelbagi, Ishag Adam

**Affiliations:** 1 Faculty of Medicine and Health Science, Gadarif University, Gadarif, Sudan; 2 Royal Commission Hospital at AL Jubail Industrial City, Al Jubail, Kingdom of Saudi Arabia; 3 Department of Pathology, AL Qunfudhah Faculty of Medicine, Umm Al-Qura University, Al Qunfudhah, Saudi Arabia; 4 Department of Obstetrics and Gynecology, Unaizah College of Medicine and Medical Sciences, Qassim University, Unaizah, Saudi Arabia

**Keywords:** Prevalence, Erectile dysfunction, Associated factors, Diabetes, Sudan

## Abstract

**Background:**

Diabetes mellitus (DM) is a global health threat and burden that is associated with many chronic complications. Erectile dysfunction (ED) among patients with DM is one of these complications. There is no published data on ED in Sudan; hence, we conducted this study to evaluate the prevalence of ED and the associated factors in patients with type 2 diabetes mellitus (T2DM) in eastern Sudan.

**Methods:**

We performed a cross-sectional study. Data on blood glucose level, cholesterol level, anthropometric and demographic characteristics, results of the International Index of Erectile Function (IIEF-5) questionnaire, and clinical history were obtained.

**Results:**

A total of 334 men with T2DM with a median (interquartile range [IQR]) age of 55.0 (±10.0) years were enrolled in the study. The median (IQR) of the duration of DM was 7.0 (±8) years, and 260 (77.8%) had uncontrolled T2DM. The median (IQR) body mass index was 24.5 (±4.9) kg/m^2^.

Of the participants, 81 (24.3%) had severe ED, 52 (15.6%) had moderate ED, 75 (22.5%) had mild to moderate ED, and 63 (13.9%) had mild ED. Of the 334 men, 271 (81.1%) had ED. Logistic regression analysis showed that age (adjusted Odds Ratio [AOR] = 1.07, 95% confidence interval [CI] = 1.01–1.14), duration of DM (AOR = 1.26, 95% CI = 1.06–1.51), and cholesterol levels (AOR = 3.53, 95% CI = 1.75–7.11) were positively associated with ED. Moreover, poor glycaemic control (AOR = 3.38, 95% CI = 1.70–6.71, *P* < 0.001) was significantly associated with ED.

**Conclusion:**

There is a high prevalence of ED among patients with T2DM in eastern Sudan. Age, duration of DM, and cholesterol were positively associated with ED.

## Introduction

The incidence of diabetes mellitus (DM) is rising globally. Specifically, global prevalence in 2019 was estimated to be 9.3% (463 million people) and is expected to increase to 10.2% and 10.9% by 2030 and 2045, respectively. The majority (90%) of patients with DM have type 2 DM [1, 2]. Moreover, the estimated number of people aged 20–79 years who will be vulnerable to the disease is expected to rise to 700 million by 2045 [1, 2].

Erectile dysfunction (ED) is one of the most common complications among patients with DM, but it remains underestimated in this group. ED is the inability to achieve and sustain an erection sufficient to perform satisfactory sexual intercourse [3]. Some African countries have reported a higher prevalence of ED [4, 5]. ED is a multifactorial and complex disorder that is associated with certain risk factors in patients with DM, such as poor glycaemic control, longer duration of DM, obesity, older age, smoking, alcohol consumption, housebound bedridden status, and sedentary work [4, 6, 7]. Likewise, rural and urban residence status has an influence on ED among patients with DM [8–10]. Generally, ED contributes to a poorer quality of life in patients with DM [11].

The International Diabetes Federation’s diabetes atlas published in 2019 grouped Sudan among those countries that have a prevalence of DM of more than 12% [11]. This is consistent with a recent study from Sudan that documented relatively higher prevalence rates of T2DM and uncontrolled T2DM, which were 20.8% and 80.0%, respectively [12]. There is no published data on ED among patients with T2DM in Sudan. Hence, we conducted this study to evaluate the prevalence of ED and its associated factors in patients with T2DM in eastern Sudan.

## Study design and participants

### Study area

Gadarif is one of the 18 states of Sudan that is located in the eastern part of the country and has an area of 75,263 km^2^. The estimated population is approximately 1,348,378 people (25% urban, 73.7% rural, and 1.3% nomadic). The mosaic of the population represents the Sudanese tribal variety that practices agricultural and pastoral activities [13].

### Subjects and study design

This study was conducted to evaluate the prevalence of ED among patients with T2DM. Patients with T2DM were recruited from outpatient diabetic clinics in the Gadarif Diabetic Centre. The Gadarif Diabetic Centre is located in the centre of the city of Gadarif, which provides outpatient care services to all patients with DM in the state.

### Inclusion criteria

Married men who were known to have type 2DM, aged 30–69 years, capable of signing informed consent, and without physical disabilities preventing them from anthropometric evaluation were eligible for the present study.

### Exclusion criteria

Unmarried men or those with known secondary ED from genetic, endocrine, neurological, or surgical causes were excluded from the study. Those who were below the age of 30 years, patients with type 1 DM, and those who refused to participate were also excluded. Also, individuals with ED before the diagnosis of DM and those not engaging in any form of sexual activity at the time of the interview were excluded.

### Sample size calculation

A single population proportion formula was used to calculate the sample size. We assumed that 68% of men would have ED. There is no data on ED in Sudan; thus, this assumption was guided by the prevalence of ED in Ethiopia, which is 69.9% [14]. Thus, the sample size of 334 men was determined with a 95% confidence level, and we expected that 10% might not respond.

### Sampling procedure

A systematic random sampling method was used. There were around 1850 men (from the records of the clinic) who presented to the clinic in the previous three months. The interval was around five, which was reached by dividing the number of men (1850) by the sample size (334).

### Data collection and analysis

Two trained general practitioners under direct supervision of the team collected the sociodemographic characteristics through face-to-face interviews using a questionnaire. The sociodemographic characteristics were age, weight, height, alcohol consumption (never, current, or former), smoking (never, current, or former), comorbidities (hypertension, ischaemic heart disease, bronchial asthma, rheumatoid arthritis, and others), and presence of peripheral neuropathy. The questionnaire was also used to document a detailed history regarding DM, which included the duration of DM, number of medications, insulin therapy, regular follow-up, presence of comorbidities, complications related to DM, and current status. Laboratory tests for HBA1C and total cholesterol were obtained. Also peripheral neuropathy was tested in the foot care unit.

The participants’ weight and height were measured using standard procedures, and body mass index (BMI) was computed using the equation of weight (kg)/height (m^2^) [15].

ED was assessed by using a questionnaire (Arabic translated version) adopted from the abridged five-item version of the International Index of Erectile Function (IIEF-5) score [16]. The following operational definitions were used for the outcome variable of ED based on the scores of the IIEF-5.

**Severe ED**: Study participants who scored 5–7 out of 25 points.

**Moderate ED**: Study participants who scored 8–11 out of 25 points.

**Mild to Moderate ED**: Study participants who scored 12–16 out of 25 points.

**Mild ED:** Study participants who scored 17–21 out of 25 points.

### Measurement of HbA1C

A total of 3 ml of venous blood was drawn from each participant, preceded by a full explanation regarding the procedure and technique. Then, the site of the puncture for the blood sample was adequately disinfected by alcohol swab. The blood was then extracted into a vacuum blood collection tube containing ethylene diamine tetra acetate (EDTA), after which the sample was transferred to the laboratory department of the diabetic centre to measure the HbA1c levels using an I Chroma machine (Republic of Korea). Glycaemic control was defined in accordance with the specifications of the American Diabetes Association for non-pregnant adults and the International Diabetes Federation [1], as follows: Good glycaemic control was determined when the HbA1c target was < 7.0%, and glycaemic control was considered uncontrolled if HbA1c levels were ⩾ 7.0%.

### Standardised definitions for dyslipidaemia

Blood samples were taken after a 12-hour fast (no food or drink, except water) to assess total cholesterol. We adopted the National Cholesterol Education Program/Adult Treatment Panel III (NCEP/ATP III) definition in the Third Report of the Expert Panel on Detection, Evaluation, and Treatment of High Blood Cholesterol in Adults [17] as a reference for the diagnostic criteria for high cholesterol:

- Total cholesterol > 5.17 mmol/l (> 200 mg/dl).

### Statistical analysis

Data were analysed with a computer using SPSS for Windows (version 20.0). Continuous data were checked for normality with the Shapiro–Wilk test and were found not to be normally distributed. Data were expressed as proportions or as medians (IQR). Univariate analysis was performed with ED as the dependent variable. Independent variables included age, BMI, lipid profile, smoking (never or current/former), alcohol consumption (never or current/former), diabetes mellitus duration, glycaemic control (control or not control), number of medications, diabetes-related complications, and associated comorbidities. Multicollinearity (variance inflation factor < 4) was checked for but not detected. Variables were shifted to the logistic regression analyses if their univariate *p* was < 0.20 and backward-stepwise likelihood ratio regression was used for adjustment. Odds ratios (ORs) and 95% confidence intervals (CIs) were calculated, and a *p* value of < 0.05 was considered significant.

## Results

### General characteristics

A total of 334 men with T2DM were enrolled in the study. The median (IQR) of the age and duration of diabetes was 55.0 (10.0) years and 7.0 (8.0) years, respectively. Of these men, 260 (77.8%) had uncontrolled DM, and 102 (30.5%) had comorbidities. Twelve (3.6%) men had cardiovascular accidents, 11 (3.3%) had ischaemic heart disease, 11 (3.3%) had neuropathy, 30 (9.0%) had diabetic foot, and 38 (11.37%) had retinopathy. Of these men, 55 (16.5%) had high cholesterol, and 166 (49.7%) had regular follow-up. The median (IQR) number of medications was 2 (1). Of the 334 men, 212 (63.5%) were urban residents, 161 (48.2%) were employees, 152 (45.5%) had an education level ≥ secondary level, and 139 (41.6%) had high/moderate income. The majority of the participants, 314 (94.0%), had never consumed alcohol, and 259 (77.5%) had never smoked. The median (IQR) BMI was 24.5 (4.9) kg/m^2^ (Table 1).

**Table 1:** Sociodemographic characteristics of men with type 2 diabetes mellitus in eastern Sudan, 2020

Variables			
		**Median**	**Interquartile range**
**Age, years**		55.0	10.0
**Body mass index, kg/**^**2**^**m**		24.5	4.9
**Duration of diabetes**		7.0	8.0
**Number of medications**		2	1
		**Number**	**Percentage**
**Employment**	Yes	161	48.2
	No	173	51.8
**Education level**	≥ secondary level	152	45.5
	< secondary level	279	54.5
**Presence of co-morbidity**	No	232	69.5
	Yes	102	30.5
**Alcohol**	Never	314	94.0
	Current/former	20	6.0
**Smoking**	Never	259	77.5
	Current/former	75	22.5
**High cholesterol**	No	279	83.5
	Yes	55	16.5
**Follow-up**	Regular	166	49.7
	Not regular	168	50.3
**Residence**	Urban	212	63.5
	Rural	122	36.5
**Income**	High/ moderate	139	41.6
	Low	195	58.4
**Housing**	Family		
	Friend		
**Diabetes**	Control	74	22.2
	Not control	260	77.8

Of the participants, 271 (81.1%) had ED. Of those with ED, 81 (24.3%) men had severe ED, 52 (15.6%) had moderate ED, 75 (22.5%) had mild to moderate ED, and 63 (13.9%) had mild ED. There was no significant difference in employment, income, educational level, residence, BMI, smoking, alcohol consumption, comorbidities, or number of medications between men with ED and those without ED. The patients with ED had a significantly older age, a longer duration of DM, uncontrolled DM, and high levels of cholesterol (Table 2).

**Table 2:** Factors associated with erectile dysfunction in eastern Sudan, 2020

Variables		Erectile dysfunction (number =271)	No erectile dysfunction (number= 63)			
				**OR**	**95% CI**	***p*-value**
***Median (interquartile range***						
**Age, years**		57 (7)	55 (11)	1.10	1.04–1.15	<0.001
**Body mass index, kg/**^**2**^**m**		23.6 (4.9)	24.7 (5.1)	0.95	0.89–1.01	0.098
**Duration of diabetes**		11.0 (9.0)	7.0 (7.0)	1.10	1.05–1.15	<0.001
**Number of medications**		2 (1)	2 (1)	1.05	0.83–1.33	0.631
**Hemoglobin a 1c**^**a**^		10.4 (2.3)	8.9 (3.5)	1.39	1.21–1.59	<0.001
**Cholesterol**^**a**^		176.0 (100.0)	140 (41.0)	1.01	1.01–1.02	<0.001
*Number (percentage*						
**Employment**	Yes	134 (49.4)	27 (42.9	Reference		
	No	137 (50.6)	36 (57.1)	0.76	0.44–1.33	0.347
**Education level**	≥ secondary level	129 (47.6)	23 (36.5)	Reference		
	< secondary level	142 (52.4)	40 (63.5)	0.63	0.36–1.11	0.133
**Presence of co-morbidity**	No	188 (69.4)	44 (69.8)	1.02	0.56–1.85	0.989
	Yes	83 (30.6)	19 (30.2)	Reference		
**Alcohol**	Never	254 (93.7)	60 (95.2)	Reference		
	Current/former	17 (6.3)	3 (4.8)	1.33	0.38–4.71	0.650
**Smoking**	Never	212 (78.2)	47 (74.6)	Reference		
	Current/former	59 (21.8)	16 (25.4)	1.22	0.64–2.31	0.535
**Follow-up**	Regular	141 (52.0)	25 (39.7)	Reference		
	Not regular	130 (48.0)	38 (60.3)	0.60	0.34–1.06	0.093
**Residence**	Urban	176 (64.9)	36 (57.1)	Reference		
	Rural	95 (35.1)	26 (42.9)	0.72	0.41–1.25	0.249
**Income**	High/ moderate	116 (42.8)	23 (36.5)	Reference		
	Low	155 (57.2)	40 (63.5)	0.76	0.43–1.35	0.362
**Diabetes**^**a**^	Controlled	50 (18.5)	24 (38.1)	Reference		
	Uncontrolled	221 (81.5)	39 (61.9)	2.72	1.50–4.92	0.001

^**a**^Were entered one by one in the model

*OR* Odd ratio

*CI* Confidence interval

The logistic regression analysis showed no significant associations between education level, BMI, or being on regular follow-up. However, age (AOR = 1.07, 95% CI = 1.01–1.14), duration of DM (AOR =1.26, 95% CI 1.06–1.51), and cholesterol levels (AOR =3.53, 95% CI =1.75–7.11) were significantly associated with ED. Moreover, poor glycaemic control (AOR = 3.38, 95% CI = 1.70–6.71) was significantly associated with ED (Table 3).

**Table 3:** Factors associated with erectile dysfunction in eastern Sudan, 2020

Variables		Non-adjusted values			Adjusted values		
		**OR**	**95% CI**	***p*-value**	**OR**	**95% CI**	***p*-value**
**Age, years**		1.09	1.03–1.16	0.002	1.07	1.01–1.14	0.009
**Body mass index, kg/**^**2**^**m**		1.05	0.98–1.12	0.114	1.05	0.98–1.13	0.114
**Duration of diabetes**		1.05	1.01–1.10	0.042	1.26	1.06–1.51	0.009
**Hemoglobin a 1c**^**a**^		1.26	1.06–1.51	0.009	1.01	1.01–1.02	<0.001
**Cholesterol**^**a**^		1.01	1.002–1. 01	0.010	1.01	1.002–1. 01	0.010
**Education level**	≥ secondary level	Reference					
	< secondary level	0.57	0.30–1.07	0.085	0.57	0.30–1.07	0.085
**High cholesterol**^**a**^	No	Reference					
	Yes	3.37	1.66–6.85	0.001	3.53	1.75–7.11	0.001
**Follow-up**	Regular	Reference					
	Not regular	0.98	0.51–1.88	0.950			
**Diabetes**^**a**^	Control	Reference					
	Not control	3.48	1.73–7.00	<0.001	3.38	1.70–6.71	<0.001

^**a**^Were entered one by one in the model

*OR* Odd ratio

*CI* Confidence interval

## Discussion

The main finding of this study was the very high prevalence (81.1%) of ED among patients with T2DM in eastern Sudan. This was considerably higher than the pooled prevalence of ED reported in patients with DM in two recently published systematic reviews and meta-analyses, one of which was in Africa, with a reported rate of 71.45%, (3,501 participants) [18], and the other of which was restricted to Ethiopia, with a rate of 54.3%, (2003 participants) [19]. Globally, a meta-analysis and literature review showed that the pooled prevalence of ED among patients with DM was 57.7% [19], and 35% to 90%, respectively [20]. Our results show that the prevalence of ED among patients with T2DM is higher than those documented in different African countries, including northern Ethiopia at 69.9% [14], South Africa at 77.1% [9], Ghana at 67.9%, and Tanzania at 29.7% [21]. At the same time, the prevalence of ED in our study was lower than that among patients with T2DM in Nigeria (94.7%) [4] and Northwest Ethiopia (85.5%) [22]. The high prevalence of ED in these developing countries may be in concordance with the fact that the magnitude of ED is usually underestimated in many developing countries [8]. Further, the higher prevalence of ED obtained in this study may be linked to the global rise of DM in high, middle, and low income countries [23]. In addition, delayed detection and management of risk factors have been shown to contribute to the development of ED [8]. Alternatively, the variation in prevalence of ED obtained in different studies might be due to differences in the adopted methodology and population characteristics [18, 20, 24], as well as the variation in health-seeking behaviours between the populations [18]. ED has also been underestimated in many developing countries, including African ones, because it is not a life-threatening disorder and associated with a certain stigma; hence, men with such a problem rarely seek treatment [8].

Our study showed that age was positively associated with ED. A similar significant association between ED and old age among patients with DM was demonstrated in two other studies, including a systematic review and meta-analysis in Africa [18] and a literature review [24]. Also, it was reported in different countries across the globe, including Ethiopia [14, 19, 22, 25], Nigeria [4], Bangladesh [6], Northern Sri Lanka [7], Korea [26] and China [27].

In the present study, the duration of T2DM was positively associated with ED. This was supported by a similar outcome that was documented in a systematic review and meta-analysis assessing male patients with DM in Africa [18], as well as a literature review from Tanzania [24]. Likewise, a longer duration of DM was found to be a significant predictor for ED in many studies conducted in Ethiopia [14, 19, 25], Nigeria [4], Bangladesh [6], Northern Sri Lanka [7], Korea [26], and China [27]. This significant association of old age and duration of DM among these patients is considered a potential risk for developing secondary ED as a result of angiopathic, neuropathic, and myopathic damage complicating the primary disease [28]. Moreover, the pathophysiology of ED in DM involves multiple different mechanisms related to the primary disease (DM), including endothelial dysfunction, oxidative stress, the accumulation of advanced glycation end products, and autonomic neuropathy [29–31].

In this study, a significant association was observed between poor glycaemic control and ED among patients with T2DM. The same outcome was obtained in a similar group of patients in a literature review from Tanzania [24] and in studies conducted in Nigeria [4], Bangladesh [6], Korea [26], and China [27]. It is worth noting that hyperglycaemia in DM is a risk factor for ED, and particularly for microvascular and neuropathic complications [4, 32]. At the same time, a non-significant association between hyperglycaemia and ED was documented in some studies conducted in Ethiopia [14, 33] and Northern Sri Lanka [7].

Our study found that high levels of serum cholesterol were a significant predictor for ED among patients with T2DM. The same finding was observed in cross-sectional studies included in a literature review conducted in 2009 [24]. Likewise, similar reports demonstrated that hyperlipidaemia was a significant risk factor for developing ED among this group of patients across the globe [34–37]. In contrast to this finding, one study documented a non-significant relationship between ED and dyslipidaemia among males with DM in Sri Lanka [7]. The significant association may reflect the negative effects of hyperlipidaemia on the vascular smooth muscle tissue of the penis and the peripheral cavernous nerve [38, 39]. Moreover, hyperlipidaemia-induced impairments in erectile functions might be related to an increase in plasma asymmetrical dimethylarginine levels, changes in the regulation of the endothelial nitric oxide synthase (eNOS) levels, and eNOS expression in cavernous tissues [37]. Additionally, one study revealed that different statin types might have different effects on erectile dysfunction [40]. However, some data point to the beneficial effects of statin therapy on improving ED [36, 41], this is because it reduces endothelial dysfunction, a key etiopathogenetic factor in the onset of ED, and an element of secondary prevention for vascular events [41].

Our study showed that urban residence was not a significant risk factor for developing ED among patients with T2DM. This was supported by the outcome of two recently published articles documenting the non-significant association in a similar group of patients [42, 43]. However, a significant association was documented among males with DM in urban areas in China [10] and South Africa [9], and in a rural area in Nigeria [8]. Further, our study revealed a non-significant association with obesity and ED among patients with T2DM. Similarly, no significant association was observed in some recently published studies from Ethiopia [14, 18]. In contrast to this finding, many studies have reported a significant association worldwide between ED and increased BMI [4, 7, 44].

The present study revealed a non-significant association between ED and comorbidities related to metabolic syndrome, such as hypertension, obesity, and dyslipidaemia. In others studies with clinical data evaluating patients with DM, a significant association between comorbidities and ED was reported [6, 7, 20, 45]. Our study also showed a non-significant association between alcohol consumption and ED in subjects with T2DM, which might be explained by the small number of patients who consumed alcohol. This result was consistent with a similar finding among the same group of patients in a recently published study in Ethiopia [14]. In contrast to this finding, some studies demonstrated a significant association between alcohol intake and ED among males with DM [7, 46]. Finally, non-significant association between current smoking and ED was reported in the present study, which coincided with the results obtained for similar patients in other clinical studies [7, 14, 22, 32]. At the same time, a significant association was documented in some studies that identified smoking habits as a risk factor for ED [24, 47].

## Conclusion

There is a high prevalence of ED among Sudanese patients with T2DM. Old age, duration of DM and glycaemic control are predictors for developing ED. The high prevalence of ED in this setting is another hidden burden for patients and the health system; hence, earlier routine assessment and treatment of ED in patients with T2DM is recommended to improve the prognosis and quality of life.

### Limitations

While the present study addressed an issue that was not well studied in Sudan, some other potentially relevant factors were not evaluated, such as testosterone levels, some components of lipid profile as LDL and HDL, daily physical exercise, family history, employment, and income. The detail information about the drugs used by the patients especially antihypertensive drugs and statins was not obtained. In addition, since the study was conducted among patients in hospitals, the findings may not represent patients on follow-up in other settings.

## Data Availability

The datasets used and/or analysed during the current study are available from the corresponding author on reasonable request

## Abbreviations

AOR: Adjusted odds ratio
BMI: Body mass index
CI: confidence interval
SD: Standard deviation
ED: Erectile dysfunction
DM: Diabetes mellitus
T2DM: Type two diabetes mellitus
HbA1c: Hemoglobin A1C
IIEF-5: 5-item version of the International Index of Erectile Function
NCEP/ATP III: The National Cholesterol Education Program/Adult Treatment Panel III
eNOS: The endothelial nitric oxide synthase
EDTA: Ethylene diamine tetraacetic acid
